# Is chronic ventilatory support really effective in patients with amyotrophic lateral sclerosis?

**DOI:** 10.1007/s00415-016-8288-8

**Published:** 2016-09-16

**Authors:** A. Hazenberg, H. A. M. Kerstjens, S. C. L. Prins, K. M. Vermeulen, P. J. Wijkstra

**Affiliations:** 1 Department of Home Mechanical Ventilation, University of Groningen, University Medical Center Groningen, Groningen, The Netherlands; 2 Department of Pulmonology and Tuberculosis, University of Groningen, University Medical Center Groningen, Groningen, The Netherlands; 3 University Medical Center Groningen, GRIAC Research Institute, University of Groningen, Groningen, The Netherlands; 4 TNO, The Netherlands Organization for Applied Scientific Research, The Hague, The Netherlands; 5 Department of Epidemiology, University of Groningen, University Medical Center Groningen, Groningen, The Netherlands

**Keywords:** Amyotrophic lateral sclerosis, Quality of life, Non-invasive ventilation

## Abstract

Most patients with amyotrophic lateral sclerosis (ALS) develop respiratory insufficiency in the advanced stage of their disease. Non-invasive ventilation (NIV) is commonly regarded to be a treatment that is effective in reducing these complaints. To assess whether the effect of NIV on gas exchange and quality of life (QOL) is different in patients with ALS versus without ALS. A post hoc analysis was done with data from a previously published trial, in which all patients were instituted on NIV. Arterial blood gasses were assessed next to QOL by generic as well as disease-specific questionnaires. 77 patients started NIV: 30 with ALS and 47 without. Both groups showed significant improvements in blood gasses after 2 and 6 months. Compared to the non-ALS group, the ALS group had significantly worse scores after 6 months in MRF-28, SRI, HADS and SF-36 than the non-ALS group. This study shows that NIV improves gas exchange, both in patients with and without ALS. QOL improves markedly more in patients without ALS than in those with ALS, in whom only some domains improve. Our observation of little or no effect in ALS patients warrants a large study limited to ALS patients only.

## Introduction

Most patients with amyotrophic lateral sclerosis (ALS) develop complaints of dyspnea, fatigue, unrefreshing sleep and morning headache in the advanced stage of their disease due to respiratory insufficiency. Chronic ventilatory support is commonly regarded to be a treatment that is effective in reducing these complaints.

Several studies presented data regarding the effects of chronic ventilatory support on quality of life (QOL) in patients with ALS. Some were positive, while others produced more reservations regarding chronic ventilatory support in these patients. In 2001, in a prospective study, QOL following non-invasive ventilation (NIV) was assessed with two questionnaires, the ALS functional rating scale-respiratory version (ALSFRS-R) and the Short Form 36 (SF-36) [1]. Early intervention of NIV resulted in an improved vitality compared to standard care in this study. In 2003, Bourke et al. presented the results of a cohort study on indications and effect of NIV on QOL in ALS patients. They used the SF-36 to assess QOL in 10 participants using NIV. In this study, the use of NIV was associated with an improved QOL and survival [2]. Finally, a third cohort study on the effects of NIV on ALS patients showed that one month after starting chronic ventilatory support, both blood gasses and QOL improved [3]. In 2006, Bourke assessed the effect of NIV, QOL and survival in participants with ALS in a randomized controlled trial [4]. Their conclusion was that NIV use in patients without bulbar dysfunction was associated with an improved QOL in some domains and a longer maintained QOL above 75 % of the pre-randomization QOL assessed by SF-36. Despite the results mentioned above, Piepers et al. concluded in their review that these studies on the use of NIV in patients with ALS differ considerably in design and endpoint definitions and that well-designed randomized controlled trials are, to their opinion, not available [5].

We agree with the notion that more well-designed studies are needed on the effect of chronic ventilatory support in relation to QOL in patients with ALS. An additional point of concern is that in most studies, only the generic SF-36 has been used, while questionnaires set up specifically to assess quality of life in patients with respiratory insufficiency like the Maugeri Respiratory Failure (MRF-28) and the Severe Respiratory Insufficiency (SRI) were not used at all. Also, we believe that it is unfortunate that the previous studies did not report on outcomes like depression and anxiety, items frequently mentioned by patients with ALS.

In a randomized controlled study assessing the effect on QOL of home versus in-hospital initiation of NIV in patients with a neuromuscular disorder (NMD) or thoracic cage problem, we showed an overall improvement of QOL after the start of NIV [6]. However, in this study, the effect size in separate diagnostic groups, such as ALS, was not assessed. It was, however, unique in the sense that not only the SF-36, but both the SRI and MRF-28 were used to assess QOL, as was the Hospital Anxiety and Depression Scale (HADS). By doing so, we created a broader perspective on QOL than with only the SF-36. In the present analysis, we hypothesized that the effect of NIV on QOL in patients with ALS, as assessed by the SF-36, SRI, MRF-28 and HADS questionnaires, is different compared to patients with other reasons for need of NIV.

## Methods

A post hoc analysis was performed in all patients who started with NIV, from a previously published randomized controlled trial [6]. Patients, who had been diagnosed with chronic respiratory failure due to a neuromuscular disorder (NMD) or a thoracic cage problem, were included. The study was approved by the Medical Ethics Committee of the University of Groningen, University Medical Center of Groningen, and written informed consent was obtained from all patients. Chronic respiratory failure was defined as daytime arterial carbon dioxide pressure (PaCO_2_) >6.0 kPa (>45 mmHg). Participants started NIV at home or in the hospital in a randomized design. In line with our hypothesis and reassuringly, the results for gas exchange and QOL were not significantly different between patients who started in the hospital versus at home [7]. In the current analysis, we pooled all patients without ALS in one group and compared it to those with ALS. Gas exchange was assessed by daytime arterial blood gasses from the radial artery, and the following self-administered questionnaires were completed: SRI [8], MRF-28 [9], the SF-36 [10] and the HADS [11]. The SRI contains seven domains covering: respiratory complaints, physical functioning, attendant symptoms and sleep, social relationship, anxiety, psychological well-being and social functioning. Scores range between 0 and 100, with high sores representing better quality of life. The MRF-28 contains three domains: daily activities, cognitive function and invalidity; scores range from 0 (best) to 100 (worse). The SF-36 contains eight domains: physical functioning, role physical, bodily pain, general health, vitality, social functioning, role emotional and mental health completed with the physical and mental component summary score 0 (worse) to 100 (best) [12]. The HADS contains the anxiety and depression domain; scores range from 0 (best) tot 42 (worse).

### Statistical analyses

Independent-sample *t* tests were used to test for differences in change (∆) between groups from baseline to 6 months, and paired sample *t* tests were performed to assess the change within groups from baseline to 2 and 6 months. The level of statistical significance was set at *p* < 0.05. Statistical analyses were performed using IBM Statistics 22 (IBM, New York, USA).

## Results

**Fig. 1:**
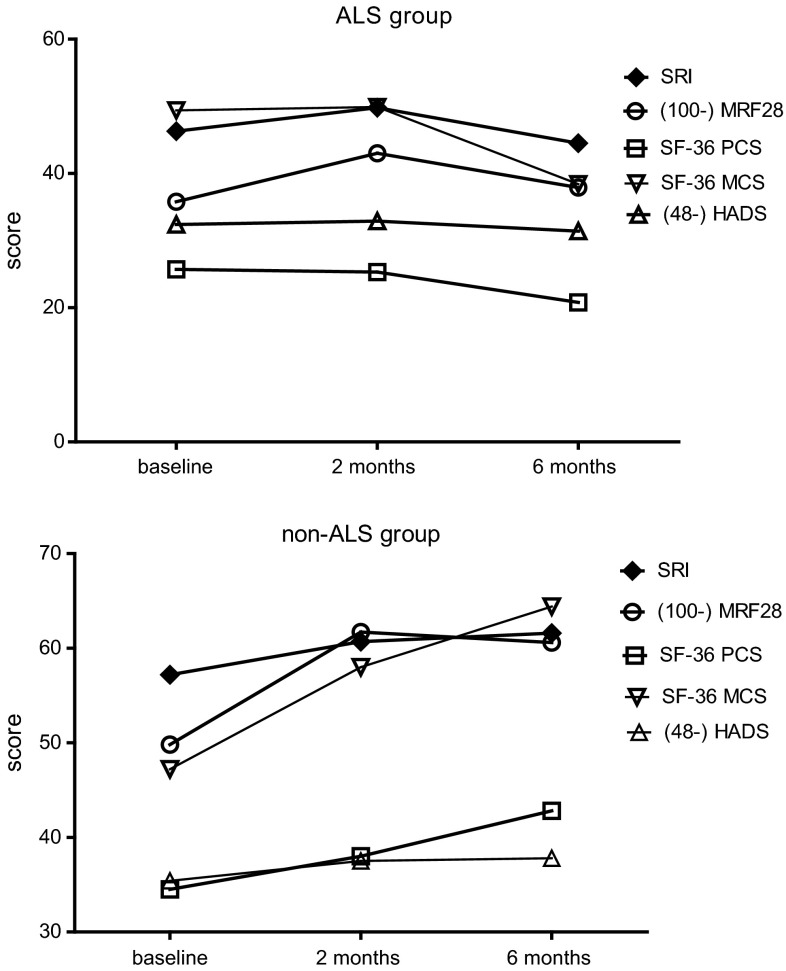
Quality-of-life measurement from baseline till 6 months after the start of chronic ventilatory support. *ALS* amyotrophic lateral scleroses, *NIV* non-invasive ventilation, *SRI* severe respiratory insufficiency (0 = worst possible health; 100 = best possible health), *MRF-28* Maugeri Respiratory Failure (0 = best possible health; 100 = worst possible health), *SF-36* Short-Form Health Status Survey (0 = worst possible health; 100 = best possible health), *SF-36 PCS* physical component score, *SF-36 MCS* mental component score, *HADS* Hospital Anxiety and Depression Scale (0 = best possible score; 48 = worst possible score)

77 participants were randomized to start NIV at home or in the University Medical Center of Groningen (UMCG). For this manuscript, both groups were pooled since there were no differences between the results in both intervention arms, as per prior hypothesis. Thirty participants had ALS (grouped as ALS), the other 47 participants had a neuromuscular disease (diaphragm paralysis, myotonic dystrophy, limb girdle muscular dystrophy, facioscapulohumeral dystrophy and other) or a thoracic cage problem (kyphoscoliosis or obesity hypoventilation syndrome) (grouped as non-ALS). The baseline characteristics are presented in Table 1 and show that both groups were comparable in age and gas exchange. NIV was initiated in the ALS group a median of 427 (22–2582) days after being diagnosed with ALS. Median survival in the ALS group after the initiation of NIV was 461 (220–1451) days; none of the participants was still alive at the moment of our investigation. Eight patients in each group withdrew during follow-up, mainly due to worsening of their disease, death or noncompliance with NIV [6]. Both groups showed an improvement in arterial and transcutaneous CO_2_ and O_2_, after 2 and 6 of NIV (Table 2); however, the mean improvements in arterial and transcutaneous CO_2_ were not significantly different between the groups. The mean improvement in PaO_2_ after 6 months was significantly lower in the non-ALS group compared to the ALS group. The mean number of hours on NIV increased over time in both groups. The ALS group used NIV for more than 11 h after 6 months and the other group 8 h per day (between group difference in change after 6 months *p* = 0.03). Some patients used NIV 24 h per day, depending on the progression of their disease. Forced vital capacity (FVC) showed a significant difference between both groups after 6 months as in the ALS group, the FVC decreased significantly, whereas the FVC in the non-ALS group improved slightly (Table 2). At baseline, quality of life was higher in the non-ALS group than in the ALS group. Compared to the non-ALS group, the ALS group showed significantly less improvement after 6 months on the MRF-28 total score. (Fig. 1; Table 3). The sum score of the MRF-28, SRI, HADS and SF-36 improved significantly in the non-ALS group after 6 months, whilst in the ALS group, only the sum score of theSF-36 improved significantly after 6 months (Table 3). QOL in the ALS group even deteriorated as expressed by the sum scores of the MRF-28, SRI and HADS, leading to significant differences between groups after 6 months.

**Table 1:** Baseline characteristics

	ALS group, *n* = 30	Non-ALS group, *n* = 47
Diagnosis	Amyotrophic lateral sclerosis	Neuromuscular disease (*n* = 42) Thoracic cage problem (*n* = 5)
Age (years)	59.6 ± 10.6	57.7 ± 14.7
Male (%)	66.7	53.2
PaCO_2_ (kPa)	6.6 ± 0.8	6.6 ± 1.0
PaO_2_ (kPa)	10.4 ± 0.9	9.6 ± 1.6
BMI (kg/m^2^)	23.8 ± 5.2	28.9 ± 6.4
Packyears	27.8 ± 19.4	18.9 ± 14.4

Data are presented as mean ± standard deviation

*kPa* kilo Pascal, *PaCO*_*2*_ partial pressure of arterial carbon dioxide, *PaO*_*2*_ partial pressure of arterial oxygen, *BMI* body mass index

**Table 2:** Changes in daytime arterial blood gasses, lung function, hours of non-invasive ventilation and transcutaneous carbon dioxide and oxygen saturation after the start of non-invasive ventilation

	ALS group			Non-ALS group			Between groups
	Baseline, *N* = 30	Change 0–2 months (*N* = 12)	Change 0–6 months (*N* = 19)	Baseline (*N* = 47)	Change 0–2 months (*N* = 17)	Change 0–6 months (*N* = 43)	*P* value for difference in change 0–6 months
PaCO_2_ (kPa)	6.6 ± 0.8	−1.2 ± 0.4*	−0.7 ± 0.3*	6.6 ± 1.0	−1.1 ± 0.2*	−0.7 ± 0.2*	0.862
PaO_2_ (kPa)	10.4 ± 0.9	1.8 ± 0.7*	0.3 ± 0.4	9.6 ± 1.6	0.3 ± 0.7*	1.3 ± 0.3*	0.035^¶^
FVC (% pred)	41.6 ± 13.7	–	−9.8 ± 3.7*	45.2 ± 16.6	–	2.5 ± 2.1	0.005^¶^
FEV1 % FVC	88.1 ± 13.8	–	−3.2 ± 3.4	76.2 ± 12.3	–	3.0 ± 2.9	0.247

	NIV initiated, *N* = 28	*N* = 27	*N* = 23	NIV initiated, *N* = 46	*N* = 43	*N* = 42	
Hours NIV	7.7 ± 3.8	3.5 ± 1.3^*^	3.8 ± 1.5^*^	6.8 ± 1.7	0.9 ± 0.4^*^	1.1 ± 0.4*	0.031
tcpCO_2_ (kPa)	6.5 ± 0.9	−1.6 ± 0.3*	−2.5 ± 0.4	6.4 ± 0.9	−1.5 ± 0.2*	−1.7 ± 0.3*	0.111
tcSpO_2_ (%)	95.9 ± 1.5	3.3 ± 0.4	3.8 ± 0.5*	95.1 ± 1.7	6.1 ± 1.0*	6.0 ± 1.1^*^	0.103

Baseline data are presented as mean ± standard deviation

Changes over time are presented as mean ± standard error of mean

*kPa* kilo Pascal, *PaCO*_*2*_ partial pressure of arterial carbon dioxide, *PaO*_*2*_ partial pressure of arterial oxygen, *FVC* forced vital capacity, *FEV*_*1*_ forced expiratory volume in one second, *%pred* % predicted, *NIV* non-invasive ventilation, *tcpCO*_*2*_ transcutaneous carbon dioxide, *tcSpO*_*2*_ transcutaneous oxygen saturation

^¶^ *p* < 0.05: for difference in change between groups 0–6 months

* *p* < 0.05 for chances within groups from baseline

**Table 3:** Changes in quality-of-life measurements after the start of non-invasive ventilation

	ALS group			Non-ALS group			Between groups
	Baseline, *N* = 29	Change 0–2 months, *N* = 26	Change 0–6 months, *N* = 22	Baseline, *N* = 47	Change 0–2 months, *N* = 41	Change 0–6 months, *N* = 43	*P* value for difference in change 0–6 months
SRI							
Respiratory complaints	42.0 ± 16.8	6.7 ± 3.0*	2.0 ± 4.2	51.9 ± 19.5	9.7 ± 2.0	7.3 ± 2.0*	0.194
Physical functioning	21.0 ± 18.4	−3.0 ± 2.8	−7.9 ± 4.5	40.0 ± 19.7	2.9 ± 2.1	1.8 ± 3.0	0.075
Attendant symptoms/sleep	49.5 ± 14.6	15.2 ± 3.4*	15.6 ± 4.1*	53.3 ± 21.3	6.5 ± 2.3	11.4 ± 2.5*	0.363
Social relationship	68.2 ± 10.2	−0.6 ± 2.1	−6.3 ± 1.7*	70.9 ± 13.8	1.2 ± 2.0	1.3 ± 2.3	0.032^¶^
Anxiety	47.4 ± 12.1	6.9 ± 2.7*	−6.4 ± 4.2	59.5 ± 21.0	4.3 ± 2.5	9.0 ± 2.6*	0.002^¶^
Well-being	51.2 ± 17.4	1.8 ± 2.6	0.4 ± 2.8	63.5 ± 16.6	1.0 ± 2.0	−0.1 ± 1.9	0.899
Social functioning	44.5 ± 13.7	−2.7 ± 2.5	−7.1 ± 3.0*	61.5 ± 18.0	1.4 ± 2.0	1.1 ± 2.1	0.027^¶^
Summary score	46.3 ± 8.7	3.5 ± 1.8	−1.4 ± 2.4	57.2 ± 14.0	3.9 ± 1.3*	4.5 ± 1.6*	0.037^¶^
MRF-28							
Daily activities	74.0 ± 24.9	4.1 ± 2.4	5.4 ± 5.7	51.8 ± 29.2	−8.7 ± 3.6	−7.4 ± 4.3	0.083
Cognition	34.5 ± 33.0	−9.6 ± 6.2	−4.1 ± 6.2	37.8 ± 34.9	−9.7 ± 4.6*	−7.6 ± 4.8	0.675
Invalidity	73.1 ± 31.7	−2.3 ± 6.0	2.7 ± 5.8	44.2 ± 35.1	−2.9 ± 5.0	4.2 ± 5.5	0.867
Total score	64.2 ± 17.9	−4.7 ± 2.4	−1.4 ± 4.2	50.2 ± 22.1	−13.0 ± 2.9*	−11.9 ± 2.9*	0.041^¶^
HADS							
Depression	8.3 ± 7.2	−0.2 ± 0.6	1.0 ± 0.6	6.3 ± 3.8	−1.3 ± 0.4*	−1.6 ± 0.4*	0.001^¶^
Anxiety	7.3 ± 4.3	0.2 ± 0.6	0.8 ± 0.7	6.3 ± 4.0	−1.2 ± 0.5*	−1.1 ± 0.4*	0.017^¶^
Total score	15.6 ± 7.2	−0.0 ± 1.1	1.8 ± 1.1	12.6 ± 7.2	−2.5 ± 0.8*	−2.7 ± 0.7*	0.001^¶^
SF-36							
Physical functioning	10.3 ± 17.6	−1.0 ± 1.4	−7.1 ± 3.2*	18.2 ± 21.5	4.0 ± 1.3*	4.4 ± 2.0*	0.002^¶^
Role physical	6.0 ± 12.7	1.9 ± 4.6	−4.8 ± 3.3	19.7 ± 34.9	6.1 ± 6.0	17.4 ± 7.0*	0.036^¶^
Bodily pain	66.9 ± 25.4	−3.8 ± 5.0	−11.4 ± 7.4	64.8 ± 32.5	−0.3 ± 4.4	3.0 ± 4.1	0.071
General health	19.6 ± 13.6	−0.3 ± 3.1	−3.0 ± 2.8	37.2 ± 22.4	2.5 ± 2.6	7.6 ± 3.1*	0.031^¶^
Vitality	33.6 ± 17.3	3.1 ± 3.9	4.3 ± 4.0	40.4 ± 21.4	10.6 ± 3.1*	13.3 ± 2.7*	0.065
Social functioning	44.4 ± 27.7	2.9 ± 6.3	−13.6 ± 5.9*	59.8 ± 26.5	4.6 ± 3.7	11.3 ± 4.0*	0.001^¶^
Role emotional	59.8 ± 45.7	−1.3 ± 12.8	−14.3 ± 8.5	46.1 ± 51.3	13.0 ± 9.4	20.9 ± 9.1*	0.017^¶^
Mental health	64.4 ± 20.4	−0.5 ± 2.4	0.5 ± 3.2	66.2 ± 19.4	6.0 ± 2.0*	8.6 ± 2.5*	0.050
Physical component summary	25.7 ± 10.2	−0.8 ± 2.3	−6.8 ± 2.4*	34.5 ± 16.7	3.1 ± 2.1	8.1 ± 2.8*	0.001^¶^
Mental component summary	49.4 ± 28.2	1.0 ± 7.6	−9.5 ± 5.3	47.2 ± 31.8	10.3 ± 5.6	16.6 ± 5.5*	0.004^¶^

Baseline data are presented as mean ± standard deviation

Change over time are presented as mean ± standard error of mean

^¶^ *p* < 0.05 for difference in change between groups 0–6 months

* *p* < 0.05 for changes within groups from baseline

*SRI* Severe Respiratory Insufficiency (0 = worst possible health; 100 = best possible health), *MRF-28* Maugeri Respiratory Failure (0 = best possible health; 100 = worst possible health), *HADS* Hospital Anxiety Depression Scale (0 = best possible score; 42 = worst possible score), *SF-36* Short-Form Health Status Survey (0 = worst possible health; 100 = best possible health)

Both groups showed significant improvements on the attendant symptoms and sleep domain of the SRI which improved significantly in both groups. Within the non-ALS group, many domains improved significantly after both 2 and 6 months. By contrast, in the ALS group, only three domains of the SRI improved significantly after 2 and 6 months; only the attendant symptoms and sleep domain of the SRI were still significantly improved in the ALS group as compared to baseline. Two domains of the SRI, social functioning and social relationship, worsened significantly in the ALS group, showing also a significant difference between both groups.

## Discussion

In this study, NIV was effective in improving gas exchange in both the ALS and the non-ALS group after 2 and 6 months. While NIV also clearly improved QOL in the non-ALS patients, the patients with ALS showed a different pattern. After 2 months, only three domains of the SRI questionnaire improved significantly. More importantly, quality of life became even worse in patients diagnosed with ALS as compared to the non-ALS group after 6 months of NIV.

More than 150 patients start chronic ventilatory support every year at the Department of Home Mechanical Ventilation of our hospital. Patients visit the outpatient clinic before starting chronic ventilatory support. Until now, we advised ALS patients to start chronic ventilatory support mainly based on the premise of a longer maintained QOL or even improved QOL in the randomized controlled trial of Bourke [13]. While different assessments on different moments were used, our study showed that after 2 months of NIV, QOL was maintained above 75 % of baseline in both groups. After 6 months, the domains physical functioning, role physical and role emotional of the SF-36 and physical functioning and social functioning of the SRI were under 75 % of baseline in the ALS group. Our group differs from that of Bourke et al., in that patients in the Bourke study had higher vital capacity and lower starting carbon dioxide at baseline compared to our study. In the Bourke study, there was a mean of 710 days after the first onset of weakness in any muscle before starting NIV. This suggests a similar moment of starting NIV in both studies, as the mean before the start of NIV in our study was 686 days. Our limited results in ALS were not the result of a bulbar impairment as gas exchange improved, and more importantly, only 2 out of 30 patients had a bulbar problem. Most importantly, however, we have to take into account that we did not include a control group with ALS but no NIV in contrast to Bourke, and therefore, could not assess if the NIV group was worse compared to a control group. There were also important other differences between the Bourke study and ours in the assessment of QOL. The SRI and the MRF-28 questionnaires were specifically developed for patients with respiratory failure and, therefore, used in our study, next to the HADS and SF-36. It is remarkable to see that after 6 months of chronic ventilatory support in the ALS group, only the SRI domain attendant symptoms and sleep significantly improved. In contrast, the sum score of the SRI, MRF-28 and HADS improved significantly in the non-ALS group. We think that progression of the disease ALS is one of the reasons that most domains do not improve.

Routinely, during a house call visit after a patient with ALS has died, we ask next of kin or caregivers to share their experiences with us. In general, we are told they are satisfied with the level of care and result of the therapy, comfort during sleep and more awake during daytime. However, during the last months of their life, these benefits disappear as leakage of the mask during ventilation becomes a burden for both the patient and relatives. The sound of air leaking by the mask and the ventilator wakes everybody, and does not provide any comfort anymore. We sometimes hear relatives make the remark that after the patient has died, it was a relief to see them lying in bed without mask and ventilatory support. These facts are worthwhile to consider and should be shared with patients before the start of HMV.

A limitation of our study is that it was primarily set up as a randomized controlled trial, comparing initiation of NIV at home with an in-hospital start irrespective of diagnosis and, therefore, not to compare specifically ALS to non-ALS patients. Comparing patients with and without ALS is, from a life-expectancy perspective, imperfect and should be done with caution. To understand the results of the ALS group in a broader perspective, in a future study, patients with ALS should be randomized to receive NIV or not. However, starting a randomized controlled trial, one group with NIV and one group without, is probably, from an ethical perspective, not easy to realize, because NIV has become the cornerstone of symptom management in patients with ALS.

Second, the ALS group had a smaller size than the non-ALS group resulting in lower chance of finding significant within group results compared to the non-ALS group.

In conclusion, our study shows that NIV improves blood gasses in a wide range of patients, with or without ALS. However, in patients with ALS, QOL did not improve after 6 months of NIV relative to baseline, and some domains (social functioning, social relationship and physical functioning) even showed a significant deterioration compared to baseline.

Given the doubt we create on the results of NIV in patients with ALS, we believe that prospective studies are warranted in ALS patients with proper randomized controlled setup for this question, using disease-specific QOL questionnaires.

With regard to the other studies, we think that in our study, chronic ventilatory support was initiated in a more advanced stage of the disease. Therefore, we believe that these results raise questions about the efficacy of NIV in these specific patients, but they need to be confirmed in a future dedicated randomized controlled trial in ALS patients using similar quality-of-life questionnaires.
